# Lithium intercalation mechanism into FeF_3_·0.5H_2_O as a highly stable composite cathode material

**DOI:** 10.1038/srep42237

**Published:** 2017-02-07

**Authors:** Ghulam Ali, Ji–Hoon Lee, Wonyoung Chang, Byung-Won Cho, Hun-Gi Jung, Kyung-Wan Nam, Kyung Yoon Chung

**Affiliations:** 1 Center for Energy Convergence Research, Korea Institute of Science and Technology, Hwarang–ro 14–gil 5, Seongbuk–gu, Seoul 02792, Republic of Korea; 2 Department of Energy and Materials Engineering, Dongguk University, Seoul 04620, Republic of Korea

## Abstract

The growing demand for lithium-ion batteries (LIBs) requires investigation of high-performance electrode materials with the advantages of being environmentally friendly and cost-effective. In this study, a nanocomposite of open-pyrochlore-structured FeF_3_·0.5H_2_O and reduced graphene oxide (RGO) is synthesized for use as a high-performance cathode in LIBs, where RGO provides high electrical conductivity to the composite material. The morphology of the composite shows that FeF_3_·0.5H_2_O spheres are embedded into RGO layers and high-resolution TEM image shows that those spheres are composed of primary nanoparticles with a size of ~5 nm. The cycling performance indicates that the composite electrode delivers an initial high discharge capacity of 223 mAh g^−1^ at 0.05 C, a rate capability up to a high C-rate of 10 C (47 mAh g^−1^) and stable cycle performance at 0.05 C (145 mAh g^−1^ after 100 cycles) and 0.2 C (93 mAh g^−1^ after 100 cycles) while maintaining high electrochemical reversibility. Furthermore, the responsible electrochemical reaction is investigated using *in-situ* XRD and synchrotron-based X-ray absorption spectroscopy (XAS), and the XRD results show that FeF_3_·0.5H_2_O transitions to an amorphous-like phase through a lithiation process. However, a reversible oxidation change of Fe^3+^ ↔ Fe^2+^ is identified by the XAS results.

Widespread energy consumption and environmental concerns over fossil fuels require advances in electrochemical energy storage systems ranging from portable to large-scale applications^1^,^2^,^3^. In this regard, the development of high-performance electrode materials for lithium-ion batteries (LIBs) is needed to meet the rapidly increasing demand for energy storage devices. There are plenty of anode materials which show promising electrochemical performance in LIBs such as carbonaceous materials, de/alloying based materials, metal oxides, metal oxyfluorides, etc.^4^,^5^. Unfortunately, the commercially available LIBs for portable devices have adopted cobalt-based cathode materials, which have the drawback of high fabrication costs, especially for large-scale applications^6^. Therefore, the main interest in the development of LIBs is to identify electrode materials that are cost-effective while maintaining the energy and power densities of the batteries. Metal fluorides are promising cathode materials due to their high theoretical capacities based on the multiple electron redox reaction and higher working potential than sulfides, nitrides and oxides^7^,^8^. Due to the abundance and low cost of iron, FeF_3_ is the most suitable candidate among the various types of metal fluorides. FeF_3_ exhibits high capacity (237 mAh g^−1^ for one-electron transfer and 712 mAh g^−1^ for three-electron transfer) and high working potential (>3.0 V for the Fe^2+^/Fe^3+^ redox reaction)^9^,^10^. Moreover, FeF_3_ is a better choice for Li-polymer batteries where Li metal is used as the anode^11^. Despite the high capacity and working potential, FeF_3_ is intrinsically an insulator due to the high iconic character of the metal-halogen bonds, and the poor kinetics limit its capacity^7^. High electrochemical activity can be achieved by reducing the particle size to the nanometer scale and by providing electronic conductive networks^11^,^12^. The combination of these approaches shortens the ion transport distance into the electrode and enhances the electrochemical performance of the FeF_3_ cathode^12^,^13^,^14^. As a conductive network, graphene possesses several advantages over other carbonaceous materials, such as high electric conductivity, large surface area (2630 m^2^ g^−1^), high thermal stability, and superior mechanical flexibility^15^,^16^,^17^,^18^.

The crystal structure is an important factor in the electrochemical performance of the electrode material for battery applications. FeF_3_, with or without hydrate contents, is found with several crystal structures, including cubic pyrochlore with the Fd3m space group^19^, tetragonal with the P4/n space group^20^, orthorhombic ReO_3_-type with the Cmcm space group^21^, and rhombohedral-type with the R-3c space group^22^, hexagonal tungsten bronze-type with the Cmcm space group^22^. Cubic pyrochlore FeF_3_ has the advantage of the largest unit cell volume of 1100.7 Å^3^ with large open tunnels^22^. Therefore, this FeF_3_ pyrochlore provides a high rate of alkali ion insertion/extraction. In addition, hydrated forms of iron fluorides (FeF_3_**·**xH_2_O) have recently been introduced with structural and morphological advantages^23^,^24^,^25^. FeF_3_**·**0.5H_2_O has an open pyrochlore structure (*Fd-*3*m* space group) and a large unit cell volume (1127 Å^3^), which are beneficial for the facile intercalation of alkali ions^26^,^27^,^28^. The pyrochlore-type FeF_3_**·**0.5H_2_O composite with single-walled nanotubes (SWNTs) has shown a stable capacity of 135 mAh g^−1^ in LIBs^27^. Reduced graphene oxide (RGO) has been used to increase the electrical conductivity of cathode materials that suffer from poor conductivities and to ensure a uniform distribution of particles on the surface and between the layers of graphene^29^,^30^,^31^. Furthermore, the high thermal stability of RGO helps the composites to withstand high current loads, where heat generation in the cells is significant^32^.

The aim of this study is to synthesize an RGO and FeF_3_**·**0.5H_2_O composite with the advantage of the particle size reduced to a few nanometers. The composite electrode is used as a cathode in LIBs, where its cycle and rate capability are measured under different current densities. Furthermore, the electrochemical reaction mechanism of Li^+^ insertion/extraction into FeF_3_**·**0.5H_2_O is investigated by *in-situ* x-ray diffraction (XRD) and x-ray absorption spectroscopy (XAS), which provide useful information about the structural changes.

## Results

**Figure 1:**
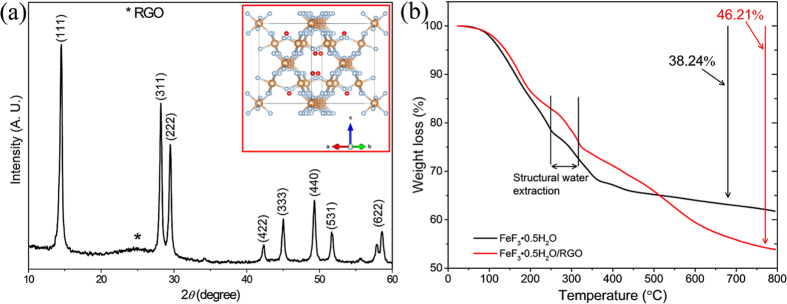
Structural characterization of FeF_3_·0.5H_2_O/RGO composite. (**a**) XRD pattern of the FeF_3_·0.5H_2_O/RGO composite (the asterisk (*) denotes the RGO peak). The inset shows the crystal structure of pyrochlore FeF_3_·0.5H_2_O, where Fe atoms are shown in gray, F atoms in blue and H_2_O molecules in red, located in a zigzag position in structure. (**b**) Thermogravimetric analysis data of the bare FeF_3_·0.5H_2_O and RGO composite.

Figure 1 shows the XRD pattern of the synthesized FeF_3_·0.5H_2_O/RGO composite. The peaks match those of pyrochlore (*Fd-3m* space group) FeF_3_**·**0.5H_2_O, and the details of its crystal structure (as shown in the inset of Fig. 1) are reported elsewhere^26^. The lattice parameter and volume of the cubic FeF_3_**·**0.5H_2_O are a = 10.405 Å and V = 1126 Å^3^, respectively. FeF_3_**·**0.5H_2_O exhibiting the open-framework structure with a large unit cell facilitates cation insertion through the 3D interconnected channels. An RGO peak can also be observed in the XRD pattern with a lower intensity (marked with asterisk (*) symbol), indicating an amorphous-like structure, and it was confirmed that the amount of RGO was ~11 wt% based on the C/S analysis. Thermogravimetric analysis (TGA) was conducted in order to observe the extraction of crystal structure water contents and to determine the amount of RGO in the composite. Figure 1b shows the TGA data of bare and composite FeF_3_**·**0.5H_2_O powders. Both the bare FeF_3_**·**0.5H_2_O and RGO composite materials show that the extraction of structural water occur in the temperature range of 248–315 °C which is comparable to literature^26^. A total weight loss of 38.14% was recorded in case of bare FeF_3_**·**0.5H_2_O at a temperature of 800 °C while it was recorded 46.21% for FeF_3_·0.5H_2_O/RGO composite. The composite material shows ~8% more weight loss compare to bare at a temperature of 800 °C which indicates the presence of carbon contents. However, this value of 8 wt.% is lower compare to the value determined by C/S analysis (11 wt.%). This is due to the fact that the bare material also contains carbon contents from the residue of ionic liquid as reported earlier^26^.

**Figure 2:**
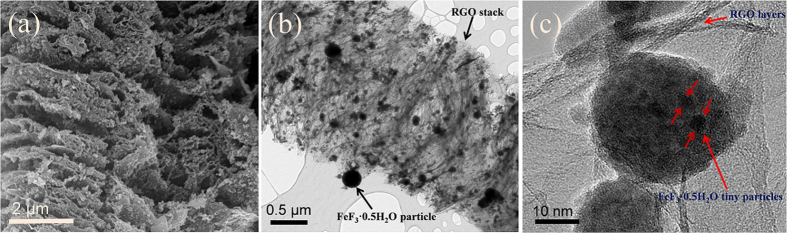
Morphological and microstructural characterizations. (**a**) SEM image of the composite. TEM images (**b**) at low and (**c**) high magnification. The red-colored arrows indicate the size of the FeF_3_·0.5H_2_O nanoparticles.

Figure 2a shows the morphology of FeF_3_**·**0.5H_2_O particles dispersed in the RGO layers, and the average size of the particles is less than 100 nm. The FeF_3_**·**0.5H_2_O particles are strongly bound to RGO. In the composite, RGO has a versatile role: RGO facilitates the electronic conduction of the composite, and its multilayer character lowers the particle-particle interface resistance because the particles are well-scattered. The uniform distribution of FeF_3_**·**0.5H_2_O particles, enabled by the introduction of RGO layers, is expected to increase the rate capability and energy storage capacity of the active material because it can provide more active sites to interact with the electrolyte. TEM imaging of the composite indicates that FeF_3_**·**0.5H_2_O particles are grown between the RGO layers, as shown in Fig. 2b. The high-magnification TEM image shows that each single particle comprises multiple nanoparticles with a size of ~5 nm, as marked by the arrows in Fig. 2c. The nanosized particles decrease the diffusion length of the cation during insertion/extraction into the structure.

**Figure 3:**
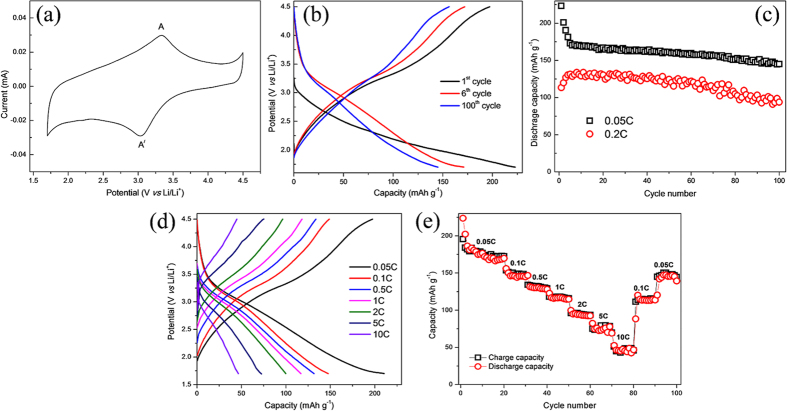
Electrochemical performance of the FeF_3_·0.5H_2_O/RGO composite cathode. (**a**) Cyclic voltammograms of the FeF_3_·0.5H_2_O/RGO composite at a scan rate of 0.2 mV s^−1^. (**b**) Potential profiles of the composite at a rate of 0.05 C for the 1^st^, 6^th^ and 100^th^ cycle. (**c**) Discharge capacity as a function of cycle number at a C-rate of 0.05 and 2 C. (**d**) Potential profiles at current rates of 0.05, 0.1, 0.5, 1, 2, 5, and 10 C. (**e**) Specific capacity versus cycle number at current rates of 0.05, 0.1, 0.5, 1, 2, 5, and 10 C.

The reaction mechanism of the composite cathode was evaluated by cyclic voltammetry (CV), and the measurements were performed at a scan rate of 0.2 mV s^−1^ over a voltage range of 1.7–4.5 V. Figure 3a shows the CV of the composite electrode and one pair of peaks corresponding to the reduction (Li insertion) and oxidation (Li extraction) peaks located at 3.03 V and 3.33 V, respectively. We believe that these value can be correlated to the average working potential during discharging and charging, respectively. The redox peak (A/A^**⁄**^) reveals the reversible reduction-oxidation of Fe (Fe^3+^/Fe^2+^) during cycling.

Figure 3b shows the galvanostatic charge/discharge profile of the composite cathode in a voltage range of 1.7–4.5 V. The first discharge curve exhibits a large capacity of 223 mAh g^−1^ at a rate of 0.05 C (1C = 220 mAh g^−1^) with a sloping potential profile. This capacity is larger than the theoretical capacity of FeF_3_**·**0.5H_2_O (220 mAh g^−1^ with one lithium insertion), presumably because of the side reactions on the surface of electrode where redox reaction or ion adsorption occurs at functional groups on the surface of ionic-liquid treated RGO^33^. The potential profile of the composite material stabilizes after a few cycles and delivers a discharge capacity of 171 mAh g^−1^ at the 6^th^ cycle, with a plateau at ~2.9 V. The obtained capacity of our cathode is superior to the literature values of ~110 mAh g^−1^ and 135 mAh g^−1^ 14,27. This stabilized value of reversible capacity (171 mAh g^−1^) corresponds to 78% of the theoretical capacity which is calculated on the basis of the active electrode material. We speculate that the electrode material cannot achieve full theoretical capacity presumably due to the presence of water molecules in the structure. The cycle performance of the material is expected to be stable as the electrode material contains water molecules which steadily forms LiOH on the surface to stabilize the reversible de/lithiation process. The cycling stability of the composite at current densities of 0.05 C and 2 C is shown in Fig. 3c. At 0.05 C, the discharge capacity is stabilized after the 5^th^ cycle, and the composite exhibits stable capacities from the 6^th^ cycle. After 100 cycles, the electrode still delivers a high discharge capacity of 145 mAh g^−1^, which corresponds to a capacity retention of 85% of the stable capacity (171 mAh g^−1^). For a cell cycled at 0.2 C, a relatively lower initial capacity of 113 mAhg^−1^ is observed. However, the delivered capacity gradually increased within the early stage of cycling and the capacity reached 129 mAh g^−1^ at the 4^th^ cycle. The increase of capacity in the initial cycles is attributed to the gradual activation of the material under the high current rate of 0.2 C. The electrode delivers a capacity of 94 mAh g^−1^ after 100 cycles, corresponding to a capacity retention of 72%.

To investigate the power performance of the composite cathodes, the charge/discharge profile as the C-rate changes from 0.05 C to 10 C is shown Fig. 3d. The composite cathode material delivered average discharge capacities of 178, 147, 132, 117, 94, 75 and 47 mAh g^−1^ at current rates of 0.05, 0.1, 0.5, 1, 2, 5 and 10 C, respectively. To the best of our knowledge, this is the first report of high rate performance for a pyrochlore FeF_3_·0.5H_2_O cathode. The active material loading and electrode thickness are important factors for electrochemical measurements and high thickness could seriously limits the Li^+^ diffusivity and rate performance^34^. The material showed excellent rate performance at high current rates, and even after deep cycling, the recovered average discharge capacity was 112 and 142 mAh g^−1^ at current rates of 0.1 and 0.05 C, respectively. Overall, the composite electrode shows good electrochemical performance, which is attributed to the nanostructure of the FeF_3_·0.5H_2_O and the facile electrolyte and electron conducting network obtained by the addition of RGO. An RGO-only electrode was assembled to observe its contribution to the capacity, and the electrodes were prepared with the same experimental conditions as for the composite material. The RGO electrode exhibits a stable discharge capacity of 95 mAh g^−1^ at rate of 0.05 C. However, because the composite electrode contains only 11 wt% RGO, it contributes negligible capacity to the composite’s performance.

**Figure 4:**
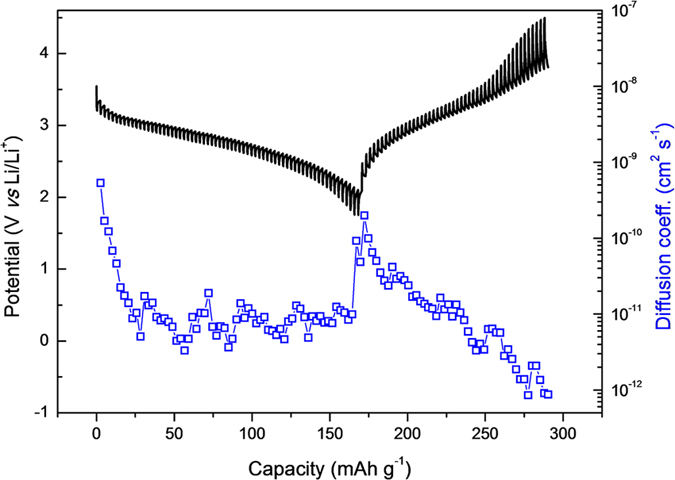
Diffusion coefficient measurement. GITT curve of the FeF_3_·0.5H_2_O/RGO composite along with the Li^+^ diffusion coefficient as a function of capacity.

The electrochemical performance of an electrode material depends on the alkali insertion/extraction process and galvanostatic intermittent titration technique (GITT) is considered as the most reliable technique to determine the Li diffusivity^35^. We performed GITT to determine the diffusion coefficient of lithium ions (D_Li_) through the composite electrode at a current density of 0.1 C within a voltage range of 1.7–4.5 V, as shown in Fig. 4. A current was applied for 10 min per each titration during discharge/charge process, with an open-circuit stand for 60 min to allow the cell potential to reach a new steady-state potential. D_Li_ was calculated by assuming that the lithium diffusion in the electrode obeys Fick’s law. The overpotential progressively increased with the discharging depth, and D_Li_ decreased from 5.3 × 10^−10^ (at the start of the discharge process) to 9.9 × 10^−12^ (at the end of the discharge process) cm^2^ s^−1^. The graph shows a large overpotential of ~3.5 V, which is the starting point of the plateau during the charge process. The D_Li_ value is 2 × 10^−10^ cm^2^ s^−1^ at the beginning and its decreases to 8.7 × 10^−13^ cm^2^ s^−1^ as the potential increases to 4.5 V. The discrepancy in the D_Li_ values at the beginning and after a complete cycle is related to the structural changes and will be explored in later sections. There is no previous report for D_Li_ in pyrochlore-structured FeF_3_·0.5H_2_O; however, these calculated values of D_Li_ are consistent with those of FeF_3_·3H_2_O, FeF_3_·0.33H_2_O, FeF_3_/C and FeF_3_/graphene^23^,^36^. Furthermore, the D_Li_ values of our composite are comparable to that of monoclinic Nb_2_O_5_ (10^−13^–10^−12^ cm^2^ s^−1^) but higher than pseudo-hexagonal (10^−17^–10^−16^ cm^2^ s^−1^) and orthorhombic (10^−15^–10^−14^ cm^2^ s^−1^) Nb_2_O_5_ phases^37^. Nano-sized layered anode materials such as Li(Li_1/3_Ti_5/3_)O_4_ has shown better lithium kinetics and the D_Li_ was calculated to be in the range of 10^−11^–10^−9^ cm^2^ s^−1^ of the cell running at 0.2 C rate^38^. The higher values of D_Li_ at the beginning of discharge/charge process indicate fast Li^+^ extraction/insertion into the nanoparticles; however, the D_Li_ values indicate more sluggish behavior with depth in the discharge/charge process, which presumably corresponds to the structural arrangement.

**Figure 5:**
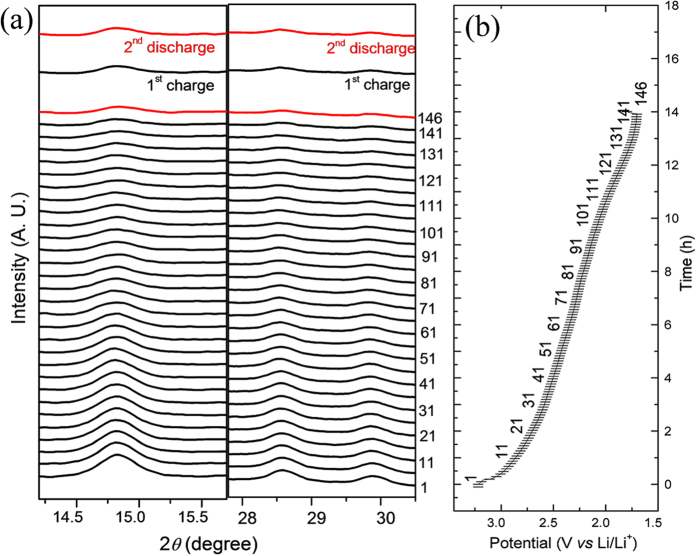
Structural investigation during lithium insertion process using *in-situ* XRD. (**a**) *In-situ* XRD of the composite electrode during the 1^st^ discharge along with the final XRD scans of the 1^st^ charge and 2^nd^ discharge. (**b**) The corresponding curve during the 1^st^ discharge process.

*In-situ* X-ray diffraction was conducted to observe the phase changes of the material during the discharge/charge process. A coin cell with a hole in the stainless steel part was used to allow the X-ray to be easily injected into the cathodes, and the cell was discharged/charged at a current density of 0.05 C over a voltage range of 1.7–4.5 V. Figure 5a shows the XRD scans in 2θ ranges of 14.2–15.7° and 27–30.5°, where the major (highest intensity) peaks are located. The corresponding discharge profile is shown in Fig. 5b. As the insertion of Li proceeds, the intensity the diffraction peaks decreases, indicating phase transformation of the material towards a poor crystalline state. No significant shift is observed in the peaks. The poor crystallinity of the material is associated with the nanosized particles and the extraction of the water contents from the structure upon Li^+^ insertion. The latter phenomenon was previously observed in the case of sodium insertion into pyrochlore FeF_3_·0.5H_2_O^26^. The lack of change in the 2*θ* values during the discharge process can be explained by assuming that Li^+^ insertion and water extraction occur simultaneously. The charge process also shows depressed diffraction peaks, and after complete charging and the 2^nd^ discharge process, the peaks are not recovered to the initial state. The irreversibility of the diffraction peaks shows that the material is not recovered after reacting with lithium. Amorphization of the material upon Li^+^ extraction/insertion is a possible cause of the capacity fade in the initial cycles, and it also causes high polarization, as observed by the GITT analysis.

**Figure 6:**
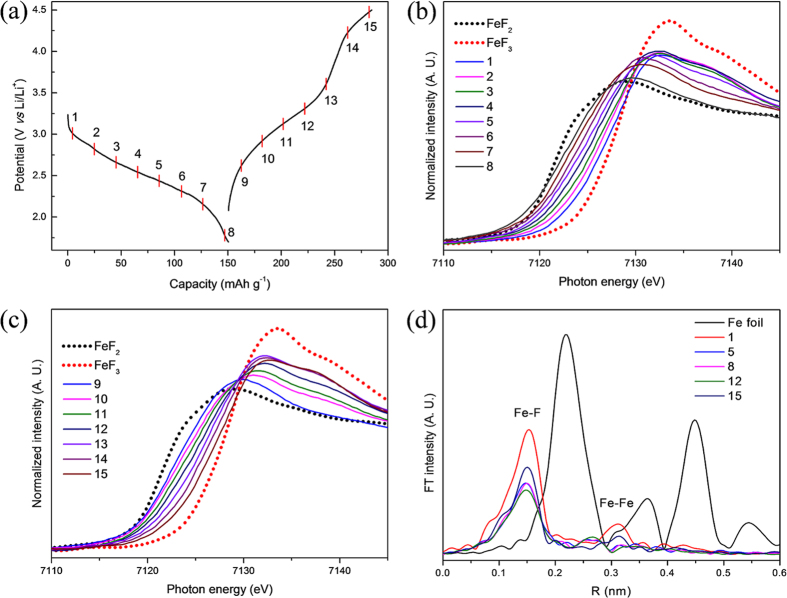
Lithium storage mechanism using *in-situ* XAS. (**a**) Potential process marked with numbers on the profile where the XANES spectra are measured. (**b**) *In-situ* XANES of the composite electrode during the discharge process and (**c**) charge process. (**d**) Selected EXAFS spectra during the discharge/charge process are plotted along with Fe metal foil.

To identify the changes in electronic structure of the composite electrode during the charging/discharging process, *in-situ* XANES analysis was performed, and the results are depicted in Fig. 6. The cell was discharged/charged at a rate of 0.1 C in the voltage range of 1.7–4.5 V, and the resulting profile is marked with number where the XANES spectra were measured (Fig. 6a). Figure 6b and c show the Fe K-edge *in-situ* XANES spectra of the composite electrode during the discharge and charge processes, respectively. The XANES spectra of the standard samples for 2+ (FeF_2_) and 3+ (FeF_3_) were also measured to observe the total valence change of Fe in the composite electrode. The absorption edge of FeF_2_ is at 7121.5 eV, and it is at a higher energy for FeF_3_ (7127 eV). The 1^st^ XANES spectrum (Fig. 6b) is at slightly lower energy than FeF_3_ because of the position of the scan on the charge-discharge curve, as shown in Fig. 6a. The XANES spectra of the composite electrode systematically shift to lower energies as the depth of discharge increases. At the fully discharged state (point 8), the edge energy (inflection point) almost overlaps that of the reference 2+, revealing the average valence state of Fe was reduced from 3+ to 2+. During the following charge process (Fig. 6c), a reversed tendency of the shift in the XANES spectra is observed, which reveals the oxidation of Fe upon the extraction of Li^+^. The XANES spectrum of the fully charged electrode (no. 15) is at slightly lower energy compared to FeF_3_ (standard), which may be due to intercalation of Li into FeF_3_·0.5H_2_O and changes in the structure, as already evidenced by the GITT and *in-situ* XRD results. Overall, the XANES results show that the capacity contribution in the composite is mainly due to the oxidation/reduction of Fe between 2+ and 3+.

To further investigate the reaction mechanism, Fourier-transformed (FT) *k*^2^-weighted Fe K-edge extended x-ray absorption fine structure (EXAFS) spectra of the selected scans (Fig. 6d) are plotted along with Fe metal foil, and the data are not phase-corrected. The first spectrum (point 1) shows two distinct peaks at approximately 0.152 and 0.311 nm, which correspond to Fe-F and Fe-Fe bonding, respectively. A significant change was observed in the intensity of the peaks during the discharge/charge process. However, no significant peak of Fe metal was observed during cycling, which suggests the reaction did not proceed through a conversion mechanism.

## Discussion

The Fe-based, low-cost, earth-abundant, environmentally benign nanocomposite showed high electrochemical performance as a cathode in SIBs, where it exhibits a high discharge capacity of 223 mAh g^−1^ in the initial cycle and 145 mAh g^−1^ at the 100^th^ cycle at a rate of 0.05 C and operates at high rate of 10 C (delivers 47 mAh g^−1^). The excellent electrochemical performance of the composite is attributed to the (i) the open-framework cubic-pyrochlore-type crystal structure, (ii) the nanostructure of the FeF_3_·0.5H_2_O, which offers a short diffusion path for Li^+^ and (iii) the incorporation of RGO, which results in high rate capabilities, indicative of the positive synergistic effect on FeF_3_·0.5H_2_O and the RGO composite. Our work shows that the composite material containing RGO and ultra-small nanoparticles with a size of ~5 nm can improve LIB performance. Furthermore, a facile co-precipitation synthesis method at room temperature makes it possible to minimize the processing costs during industrial production. This study employs a variety of methods to observe the structural evolution of the composite material during Li^+^ insertion/extraction, including GITT, *in-situ* XRD and XANES. The *in-situ* XRD results reveal that lithium insertion into the composite results in poor crystallinity of the material. The XANES study shows that Fe valence systematically changes from 3+ to 2+ during the discharge process and vice versa.

## Methods

Fe(NO_3_)_3_·9H_2_O (Aldrich Korea, 99.99%) and ionic liquid 1-butyl-3-methylimidazolium tetrafluoroborate (BMIMBF_4_) (Aldrich, >98%) were used in the synthesis of FeF_3_**·**0.5H_2_O along with reduced graphene oxide. The synthesis of FeF_3_**·**0.5H_2_O/RGO was recently reported^26^. We modified the process by reducing the amount of reduced graphene oxide and decreasing the reaction time to 8 h to obtain the uniform growth of particles with controlled size.

### Characterizations

The crystal structure of the composite was characterized with a Rigaku X-ray diffractometer with Cu-Kα radiation. The morphology of the composite was observed by scanning electron microscopy (Fe-SEM, NOVA NanoSEM200, FEI). The microstructure of the composite was observed by transmission electron spectroscopy (TEM, Tecnai G^2^ F20 FEI).

### Electrochemical tests

The electrodes were prepared by mixing the composite, carbon black and polyvinylidene difluoride (PVdF) at a weight ratio of 7:2:1. The mixture was pasted on aluminum foil, roll-pressed and vacuum-dried at 80 °C overnight. The composite electrodes with a thickness of ~30 μm and an active material weight of ~1.8 mg were used to make coin cells, and 1 M LiPF_6_ dissolved in ethylene carbonate, diethyl carbonate and dimethyl carbonate at a volume ratio of 1:1:1 was used as the electrolyte. Lithium foil was used as the counter electrode.

### *In-situ* XRD and XANES

A CR 2032 coin cell with 3 mm hole in its solid part was used for the *in-situ* XRD measurements (R–AXIS IV++, Rigaku) at the Korea Institute of Science and Technology (KIST). The cell was galvanostatically cycled at a current density of 0.05 C through a voltage range of 1.7–4.5 V using a Wonatech battery test system (WBCS 3000K8). The data were recorded with Mo–Kα radiation (λ = 0.7107 Å) and later converted to Cu–Kα radiation (λ = 1.54 Å).

XANES measurements were performed at the KIST–PAL 1D beamline of the Pohang Light Source (PLS–II), and a Si(111) double crystal monochromator was used for energy selection. The Fe K-edge (7112 eV) spectra were measured in transmission mode, and a pure iron foil was used as reference to calibrate the spectra. A Gamry instrument (Reference 600^TM^) was used for the galvanostatic cycling of the laboratory-designed coin cell, and the cell was cycled at a current density of 0.05 C over a voltage range of 1.7–4.5 V. The XANES data were analyzed using the ATHENA package^39^. It is worthy to mention that both *in-situ* XRD and XANES measurements were taken during 1^st^ galvanostatic dis/charge process.

## Additional Information

**How to cite this article**: Ali, G. *et al*. Lithium intercalation mechanism into FeF_3_·0.5H_2_O as a highly stable composite cathode material. *Sci. Rep.* **7**, 42237; doi: 10.1038/srep42237 (2017).

**Publisher's note:** Springer Nature remains neutral with regard to jurisdictional claims in published maps and institutional affiliations.
